# Transcriptional regulation by normal epithelium of premalignant to malignant progression in Barrett’s esophagus

**DOI:** 10.1038/srep35227

**Published:** 2016-10-12

**Authors:** Jia Zeng, Laimonas Kelbauskas, Aida Rezaie, Kristen Lee, Benjamin Ueberroth, Weimin Gao, Dmitry Derkach, Thai Tran, Dean Smith, Kimberly J. Bussey, Deirdre R. Meldrum

**Affiliations:** 1 Center for Biosignatures Discovery Automation, The Biodesign Institute, Arizona State University, P.O. Box 876501, Tempe, AZ 85287-6501, United States

## Abstract

In carcinogenesis, intercellular interactions within and between cell types are critical but remain poorly understood. We present a study on intercellular interactions between normal and premalignant epithelial cells and their functional relevance in the context of premalignant to malignant progression in Barrett’s esophagus. Using whole transcriptome profiling we found that in the presence of normal epithelial cells, dysplastic cells but not normal cells, exhibit marked down-regulation of a number of key signaling pathways, including the transforming growth factor beta (TGFβ) and epithelial growth factor (EGF). Functional assays revealed both cell types showed repressed proliferation and significant changes in motility (speed, displacement and directionality) as a result of interactions between the two cell types. Cellular interactions appear to be mediated through both direct cell-cell contact and secreted ligands. The findings of this study are important in that they reveal, for the first time, the effects of cellular communication on gene expression and cellular function between premalignant (dysplastic) epithelial cells and their normal counterparts.

Cell-cell interactions are essential for growth and function of multicellular organisms. Aberrant intercellular communication plays a key role in carcinogenesis and tumor progression^1^. Emerging experimental evidence demonstrates that tumors are complex biological systems of intertwined interactions and signaling with their microenvironment as opposed to merely collections of homogenous cancer cells undergoing transformation by themselves^2^. At the cellular level, carcinogenesis and progression is an ecological process involving dynamic interplays between malignant and non-malignant cells^1^. The signaling between them creates a context that promotes carcinogenesis and helps the tumor acquire the hallmark traits of cancer including acquired genomic instability and the evolution of preneoplastic cell populations with variable patterns of somatic lesions^1^,^2^.

Esophageal adenocarcinoma (EAC) is a highly lethal type of cancer with a 5-year survival rate of 14%^3^. The progression to EAC follows a sequence of events analogous to other cancers, beginning with Barrett’s esophagus (BE), followed by dysplasia of increasing degrees, and finally, adenocarcinoma^4^. Recent studies suggest that the same events linked to progression to malignancy in BE, namely elevated 4N DNA fractions, *TP53* lesions in diploid cells^5^, and an increase in clonal diversity^6^, are also associated with a wide variety of human solid tumors^7^. The Barrett’s epithelium can be safely visualized and biopsied during esophagogastroduodenoscopy. This makes BE a suitable disease model to study premalignant to malignant progression with findings potentially relevant and generalizable to other types of cancer.

Neoplastic cells in BE accumulate genetic and epigenetic alterations as they undergo evolution by natural selection. This process is influenced by surrounding cells and other factors in the microenvironment^8^. These findings suggest that cell-cell interactions in the tumor microenvironment can change epithelial cell behavior in Barrett’s esophagus.

We hypothesized that heterotypic interactions in the premalignant microenvironment can alter the gene transcription profile and progression from premalignant to malignant phenotype. Therefore, we investigated how heterotypic intercellular interactions between normal and dysplastic cells affect global gene expression profiles. We identified sets of differentially expressed genes related to cellular movement and cancer-related pathways using RNA-Seq analysis, pathway enrichment and functional assays. Notably, changes in the transcription resulting from co-culturing the two cell types were more likely to take place in dysplastic than in normal epithelial cells. We found that heterotypic interactions between normal and dysplastic cells inhibited cellular proliferation and changed motility in both dysplastic and normal cells. Normal cells were found to inhibit the growth of dysplastic cells mediated by both direct cell-cell contact and secreted ligands. Our findings suggest several signaling pathways, including TGF-β, EGF, and their downstream genes as potential targets for further studies aimed at finding biomarkers for early diagnosis, detection and risk prediction in premalignant progression of Barrett’s esophagus.

## Results

### RNA-Seq analysis of the transcriptome in esophageal epithelial normal and dysplastic cells

**Figure 1:**
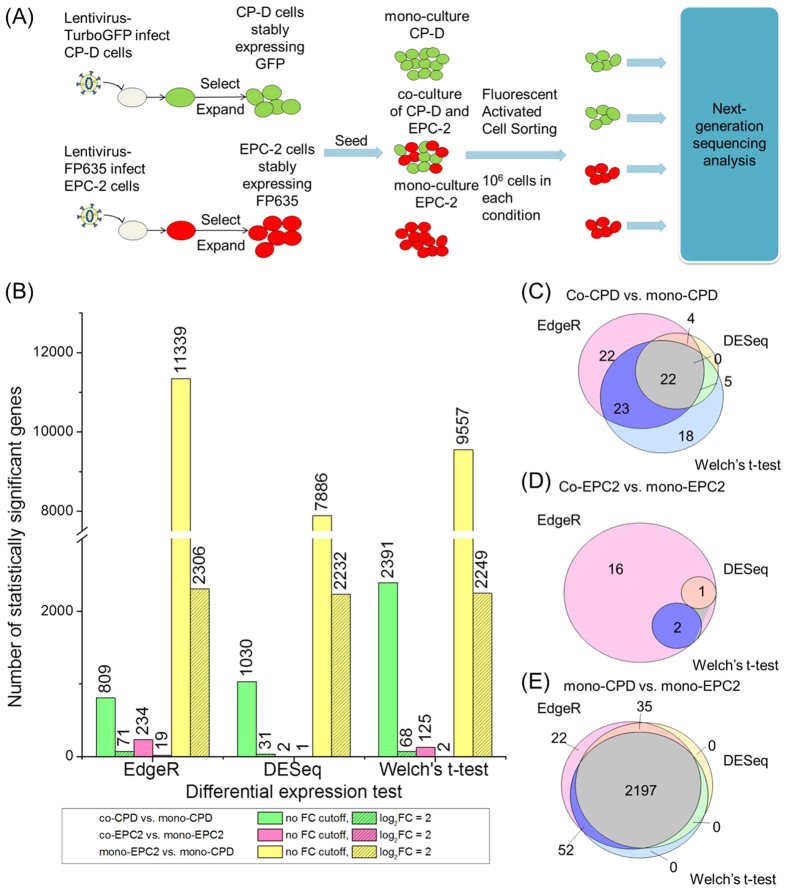
Transcriptome analysis using RNA-Seq identified differentially expressed genes in cell-cell interactions. (**A**) Workflow of transcriptome analysis of cell-cell interactions. (**B**) Number of differentially expressed genes identified using EdgeR, DESeq and Welch’s t-test, with or without fold change cutoff. (**C–E**) Venn diagram of differentially expressed genes found by different methods.

We co-cultured high-grade dysplastic cells stably expressing GFP (CP-D cell line) and esophageal epithelial squamous cells stably expressing FP635 (EPC-2 cell line) to investigate the effects of heterotypic interactions on premalignant progression in BE. Thus, cells of the two different types could be distinguished by fluorescence emission color in a culture. We used fluorescent activated cell sorting (FACS) to separate the two cell types that were then used to perform whole transcriptome sequencing (RNA-Seq) after co-culturing CP-D and EPC-2 cells for 24 hours. Mono-cultured CP-D and EPC-2 cells were used as controls (Fig. 1A). Each of the four conditions—co-cultured CP-D cells, mono-cultured CP-D cells, co-cultured EPC-2 cells and mono-cultured EPC-2 cells—contained three biological replicates. RNA-Seq was performed on an Illumina HiSeq 2000 sequencer. The majority of the 72 million reads per sample mapped to annotated gene features.

We used DESeq^9^, EdgeR^10^ and Welch’s t-test^11^ to identify differentially expressed genes in: (1) co-cultured CP-D vs. mono-cultured CP-D, (2) co-cultured EPC-2 vs. mono-cultured EPC-2, and (3) mono-cultured EPC-2 vs. mono-cultured CP-D. We performed RT-qPCR to validate selected 16 differentially expressed genes identified by Welch’s t-test in co-cultured CP-D vs. mono-cultured CP-D group and the co-cultured EPC-2 vs. mono-cultured EPC-2 group. They are the top 16 up-regulated and down-regulated genes ranked by fold change in the two comparison groups. Among the 16 analyzed genes the direction of changes in 13 genes was consistent with the RNA-Seq results (Supplementary Table S1).

The number of differentially expressed genes determined by all three methods showed the same overall trend in different pairwise comparison groups: more differentially expressed genes were found in the co-cultured CP-D vs. mono-cultured CP-D group than the co-cultured EPC-2 vs. mono-cultured EPC-2 group; the mono-cultured EPC-2 vs. mono-cultured CP-D group had the highest number among all three conditions (Fig. 1B). This indicated that gene expression profiles in dysplastic (CP-D) cells changed more due to heterotypic interactions than those in normal epithelial (EPC-2) cells. The largest difference in the number of differentially expressed genes was found in the mono-cultured CP-D vs. mono-cultured EPC-2 group.

We compared the differentially expressed genes in the three pairwise groups as determined by the different tests with FDR <0.05 and log_2_FC ≥2 (Fig. 1C–E). All three methods were concordant with each other. Particularly, a large portion of differentially expressed genes found by DESeq were also identified by EdgeR. Both EdgeR and Welch’s t-test found unique differentially expressed genes in the co-cultured CP-D and mono-cultured CP-D group, which were not shared by the other methods (genes found by all three methods are shown in Supplementary Table S2). In order to retain the ability to detect the truly differentially expressed genes with high confidence, we performed functional enrichment on differentially expressed genes identified by all three methods. However, to control for false discovery rates, further functional annotations focused mainly on the genes identified by DESeq.

### Function enrichment of differentially expressed genes in heterotypic interactions

To discover pathways related to transcriptome alterations, Ingenuity Pathway Analysis (IPA, Qiagen, Redwood City, CA) was performed to identify functional categories associated with differentially expressed genes, which were filtered by DESeq, EdgeR and Welch’s t-test independently.

**Figure 2:**
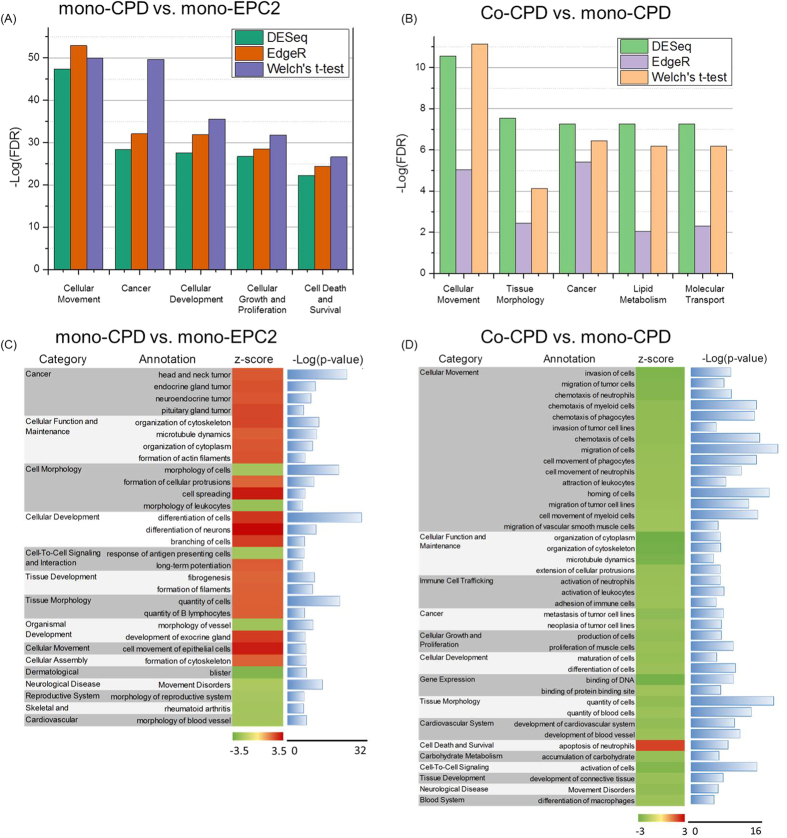
Functional enrichment analysis of differentially expressed genes. Top five functions enriched in the mono-CP-D vs. mono-EPC-2 group (**A**) and the co-CP-D vs. mono-CP-D group (**B**). The categories are sorted by –Log(FDR) from high to low based on DESeq findings. Functional annotations and predicted activation/inhibition statuses in the mono-CP-D vs. mono-EPC-2 group (**C**) and the co-CP-D vs. mono-CP-D group (**D**), threshold: activation z-score = 2, Fisher’s Exact Test.

A comparison of the differential transcription profiles of CP-D and EPC-2 cells in mono-culture showed that cellular movement, cancer, cellular development, cell growth and proliferation, and cell death and survival ranked as the top five significant biological functions (Fig. 2A). Most of them were involved in neoplasia and tumorigenesis, emphasizing that the transcriptional difference is due to the cell type difference. The majority of significantly altered functions were activated rather than inhibited (Fig. 2C). Functions involved in cancer, cellular functions and maintenance, cellular development, tissue development, tissue morphology and cellular movement were more active in CP-D cells than in EPC-2 cells, all of which may participate in neoplastic progression. Genes involved in regulating morphology were down-regulated in CP-D cells, reflecting the changes in cellular shape and size (cytology) as well as cell cohesion (architecture) and polarity in the CP-D cells.

Among the significant biological functions enriched from genes found by comparing the co-cultured CP-D to the mono-cultured CP-D group, cellular movement is among the top five functions ranked by false discovery rate (FDR, Fig. 2B). Most of the other pathways were also closely related to neoplasia and tumorigenesis, such as tissue morphology, cancer, lipid metabolism and molecular transport. We then analyzed how the biological functions overrepresented in the genes identified by DESeq method are regulated in co-cultured CP-D cells (Fig. 2D). Notably, most of the functions were suppressed in the co-cultured CP-D cells. The biggest category amongst the down-regulated functions was the cellular movement, which included a panel of movement related functions, such as invasion and migration. Other cellular movement associated functions—organization of cytoplasm, organization of cytoskeleton and microtubule dynamics—were also inhibited in co-cultured CP-D cells. Co-culturing with EPC-2 cells decreased other cancer related functions, such as metastasis and neoplasia.

In the co-cultured EPC-2 vs. mono-cultured EPC-2 group, only a few differentially expressed genes were found (Fig. 1B,D). The enrichment of most of the identified functional categories was not statistically significant.

### Upstream regulator analysis reveals inhibited TGFβ and EGF signaling in co-cultured CP-D cells

**Figure 3:**
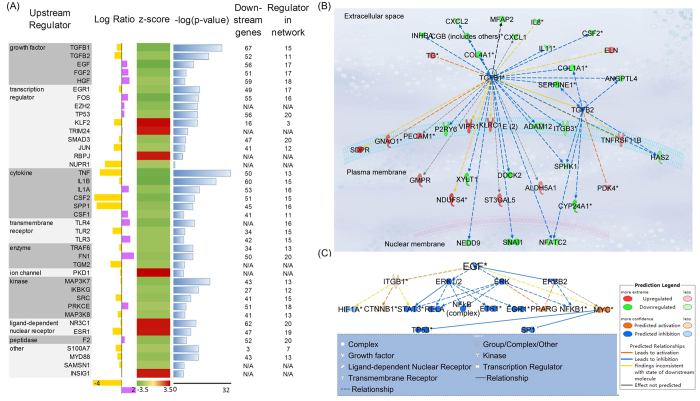
Upstream regulator analysis of the differentially expressed genes. (**A**) Upstream regulators in the co-CP-D vs. mono-CP-D group. Log ratio: Log ratio of gene expressions in each regulator. Downstream genes: downstream genes in the data set. Regulators in network: downstream regulators. The regulators were sorted based on their enrichment p-value within each category and identified regulators of pivotal importance in heterotypic interactions. (**B**) Differentially expressed genes in the network regulated by TGFβ1 and TGFβ2. (**C**) EGF mechanistic network shows the downstream regulators regulated by EGF. Depicted genes are differentially expressed.

Using IPA, we identified 40 upstream regulators and associated networks responsible for the observed transcriptional changes in the heterotypic culture condition (Fig. 3A).

Growth factors TGF-β1 (transforming growth factor β) and TGF-β2 (transforming growth factor β2) were predicted to be down-regulated in co-cultured CP-D cells. They appeared upstream of 19 regulators and affected the expression of 70 genes in their mechanistic network. There are 37 differentially expressed genes in the co-cultured CP-D vs. mono-cultured CP-D dataset which are targeted by TGF-β1 and TGF-β2. The regulation effects between these genes, TGF-β1 and TGF-β2, plotted using IPA, are shown in Fig. 3B. Among these genes, 32 were involved in epithelial neoplasia (p = 3.08E-11) and 28 in cellular movement (p = 2.24E-17).

Epidermal growth factor (EGF) was another upstream regulator predicted to be down-regulated in co-cultured CP-D cells. It affected a total of 57 differentially expressed genes in its mechanistic network according to IPA. EGF also acted upstream of 10 transcription regulators as well as seven other regulators, which were identified as differentially expressed genes in the co-cultured CP-D vs. mono-cultured CP-D comparison group (Fig. 3C). All of these regulators in the network were involved in apoptosis (p = 1.50E-15), proliferation (p = 1.95E-14), differentiation (p = 7.32E-14) and migration (p = 9.65E-14). Downstream of EGF, a panel of genes was down-regulated, including *ERBB2* and *TP53*. Increased expression of EGF receptors has been implicated in BE progression^12^ to dysplasia to adenocarcinoma. Thus, down-regulation of EGF signaling can potentially play a role in slowing down the neoplastic progression in CP-D cells.

We performed further analysis of the RNA-Seq data using DAVID^13^ and single-sample gene set enrichment analysis (ssGSEA)^14^ tools. The analysis revealed that the Wnt signaling pathway is overrepresented in the expression profiles of co-cultured CP-D cells (p-value = 3.9E-3). We found several genes of the Wnt signaling cascade to be downregulated in dysplastic cells as a result of co-culture with normal epithelial cells indicating possible suppression of Wnt-mediated signaling (Supplementary Figure S1).

### Co-culture of CP-D and EPC-2 cells changed the proliferation and motility of both cell lines

**Figure 4:**
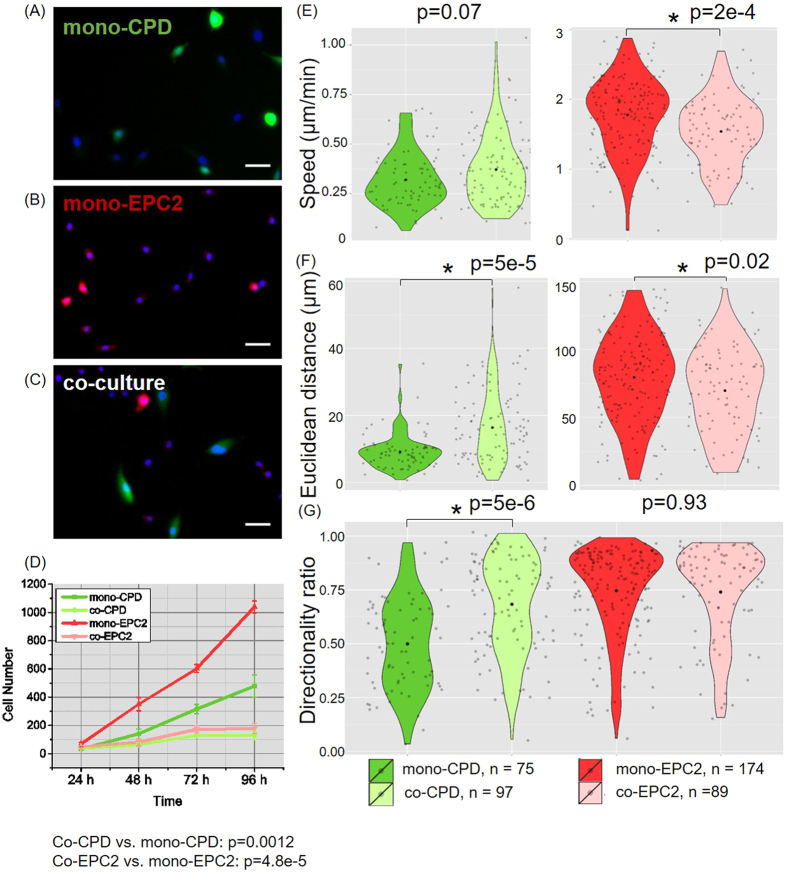
Migration and proliferation of CP-D and EPC-2 cells in mono-culture and co-culture. Fluorescence microscopy of Hoechst 33342 stained mono-CP-D cells (**A**), mono-EPC-2 cells (**B**), co-culture of CP-D and EPC-2 cells (**C**). Scale bar: 50 μm. (**D**) Proliferation of CP-D and EPC-2 cells in mono-culture and co-culture, N = 3. Slopes (k) are shown after the line. (**E–G**) Migration of CP-D and EPC-2 cells in mono-culture and co-culture. Violin plots of migration speed (**E**), displacement (**F**) and directionality ratio (**G**). *Black dot*: mean value, *grey dot*: individual data point. Mann-Whitney Test, α = 0.05.

To further investigate whether the transcriptional results corresponded to the predicted pathway outcomes (e.g. suppressed proliferation and migration), we characterized cell motility and proliferation using fluorescence microscopy. A co-culture of CP-D and EPC-2 cells seeded at a 1:1 ratio and mono-cultures of CP-D and EPC-2 cells were generated at the same cell seeding density (Fig. 4A–C, Supplementary Movies S1–3).

After counting the cells (Fig. 4D) at 24, 48, 72, and 96 hours of co-culture, we found that the proliferation of CP-D cells was slowed in the co-culture groups compared with the mono-culture groups (Table 1). Interestingly, the proliferation of EPC-2 cells in co-culture was also slower than mono-culture, although the cell growth and proliferation functional category was not significantly enriched according to the RNA-Seq analysis. This result suggests that heterotypic interaction suppressed the proliferation of both dysplastic and normal cell lines.

Cell motility was analyzed by comparing the speed, Euclidean distance and directionality ratio (Euclidean distance/displacement) for one hour after 24 hours of growth between the cells grown in co-culture vs. mono-culture. EPC-2 cells moved significantly slower in co-culture than in mono-culture (Fig. 4E). The displacement of EPC-2 cells in co-culture was significantly shorter compared with mono-culture (Fig. 4F, Supplementary Table S3). On the contrary, in co-culture with EPC-2 cells the average travel Euclidean distance of CP-D cells increased significantly compared with mono-culture CP-D cells (Fig. 4F). The co-cultured CP-D cells also exhibited significantly more directional movement than the mono-cultured CP-D cells (Fig. 4G, Supplementary Table S3).

### Conditioned media and exogenous TGF-β1 changed the proliferation and motility of both cell types

Cell-cell interactions can take place via two main types of signaling: direct cell-to-cell contact (e.g. mediated by gap junctions) or through soluble ligands in extracellular milieu by secreted cyto-/chemokines (diffusion limited, no physical contact required) or both. To gain insight into which mechanism may be responsible for the observed inhibitory effects in co-cultured cells, we have conducted a series of experiments where we investigated the effects of cell signaling via soluble ligands. To this end, the cells of each type were cultured in conditioned cell growth media obtained by swapping the media between EPC-2 and CP-D cells. Measurements of cellular proliferation and motility identical to those performed in the co-culture experiments were conducted using the same time points for measurements (24, 48, 72, 96 hours) as before. More specifically, we measured cell proliferation and motility characteristics in mono-cultures of EPC-2 and CP-D cells cultured with conditioned media produced by the corresponding other cell type, i.e. EPC-2 cells were cultured in media taken from CP-D cells and vice versa, CP-D cells were given media produced by EPC-2 cells. The results were compared against cells cultured in regular, non-conditioned media. In this way cellular communication through direct cell-cell contact was eliminated with soluble ligands being the only possible mediators.

**Figure 5:**
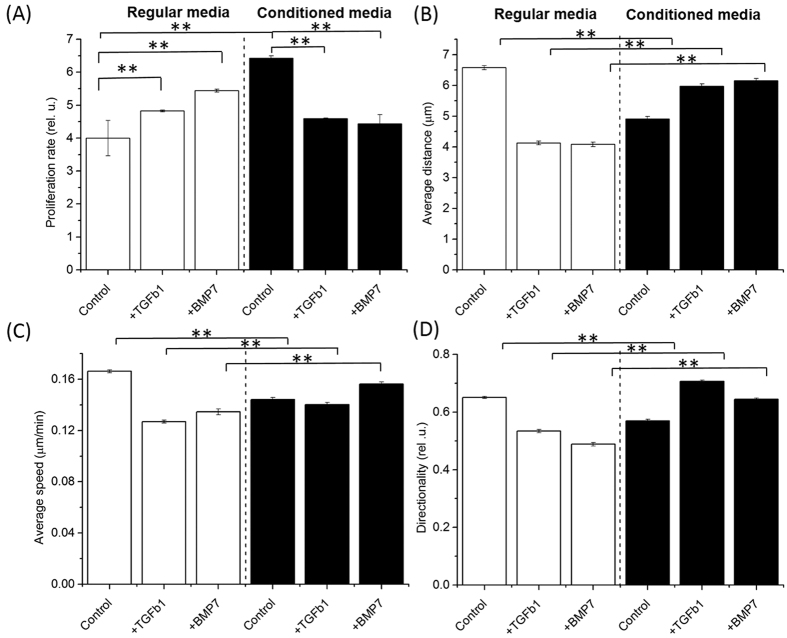
Comparison of proliferation and cell motility characteristics of dysplastic (CP-D) cells cultured in regular media and media produced (conditioned) by normal (EPC-2) cells. Proliferation rate was increased in conditioned media (Table 1), whereas all three motility parameters – distance, speed, and directionality – showed a slight downward trend (p < 0.05, Supplementary Tables S3 and S5). Compared with regular media, addition of exogenous TGF-β1 or BMP7 to conditioned media resulted in a trend reversal – a decrease in proliferation rate in conditioned compared to an increase in regular media, but an increase in all three motility parameters in conditioned vs. a decrease in regular media. Stars indicate statistical significance at p = 0.05.

**Figure 6:**
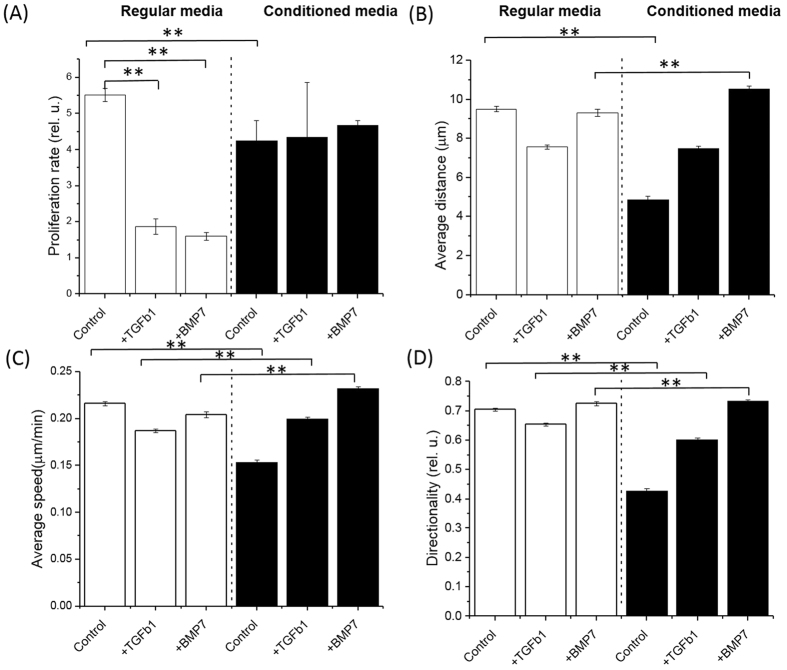
Comparison of proliferation and cell motility characteristics of normal esophageal epithelial (EPC-2) cells cultured in regular media and media conditioned by dysplastic (CP-D) cells. The proliferation rate was decreased in conditioned media (Table 1), whereas all three motility parameters – distance, speed, and directionality – showed an overall downward trend (p < 0.05, Supplementary Tables S3 and S5). The addition of exogenous TGF-β1 or BMP7 in conditioned media restored the proliferation phenotype and reversed the overall trend of the motility parameters from a slight decrease to a marked increase as compared with regular media (Table 1, Supplementary Tables S3 and S5). Stars indicate statistical significance at p = 0.05.

Notably, in contrast to the co-cultured CP-D cells (Fig. 4D), we observed a marked increase in the proliferation rate of the CP-D cells cultured in conditioned media as compared with regular media (Fig. 5A, Table 1). The EPC-2 cells showed a trend that was consistent with the finding in the co-culture experiment with a decrease in the proliferation rate in conditioned vs. regular media (Fig. 6A, Table 1). Motility analysis of both cell types revealed a shift towards lower average values of all three parameters-distance, speed, and directionality in conditioned compared to regular media and the differences are statistically significant (Figs 5B–D and 6B–D, Supplementary Tables S3 and S5). Compared to EPC-2 cells, CP-D cells exhibited lower decrease in the average values of all three motility parameters.

Next, we investigated the role of the TGF-β signaling pathway as a mediator of cellular communication between EPC-2 and CP-D cells. The pathway was identified as one of the major contributors based on the analysis of our whole transcriptome data (Fig. 3A). As a result, we studied how introduction of exogenous recombinant TGF-β1 protein into cell cultures or inhibition of the pathway may alter cellular proliferation and motility. For inhibition, we used the bone morphogenetic protein 7 (BMP7), a member of the TGF-β superfamily growth factors. BMP7 has been demonstrated to act as antagonist to TGF-β1 in epithelial-mesenchymal transition in renal injury^15^, intestinal fibrosis^16^, and EAC by inhibiting suppression of E-cadherin expression by TGF-β1^17^. In regular culture media, CP-D cells showed an increased proliferation rate in the presence of either TGF-β1 or BMP7 compared with the untreated control (Fig. 5A, Table 1). Interestingly, a reverse trend was observed for CP-D cells in conditioned media showing a slight, but statistically significant decrease in the proliferation rate in the presence of either TGF-β1 or BMP7 (p-value = 0.02 and 0.001, respectively). In contrast, addition of either TGF-β1 or BMP7 to EPC-2 cells resulted in a marked decrease in the proliferation rate in regular media, but had no significant effect in conditioned media (Fig. 6A, Table 1).

For cell motility analysis we compared the effects of TGF-β1 or BMP7 between cells grown in conditioned vs. regular media. Untreated cells in conditioned and regular media were used as controls for comparison. The motility parameter analysis revealed that in regular media in the presence of TGF-β1 or BMP7 all three characteristics of CP-D cells – distance, speed, and directionality – showed a decrease in the average values as compared with the untreated controls (Fig. 5B–D, Supplementary Figure S2). In contrast, in conditioned media, except for the speed of cells treated with TGF-β1, CP-D cells exhibited slight, but statistically significant increases of all three characteristics (Fig. 5B–D, Supplementary Figure S2, Supplementary Tables S3 and S5). A qualitatively similar behavior was also observed in EPC-2 cells (Fig. 6B–D, Supplementary Figure S3, Supplementary Tables S3 and S5). Namely, in regular media, the EPC-2 cells showed an overall decrease in all three motility characteristics. The only exception from this trend was the movement directionality which showed a small increase in EPC-2 cells treated with BMP7. Similar to CP-D cells, in conditioned media, EPC-2 cells showed increased average values of all three parameters (Fig. 6B–D, Supplementary Tables S3 and S5).

### Effect of cell-cell communication in HME1 and MDA-MB-231 co-culture

**Figure 7:**
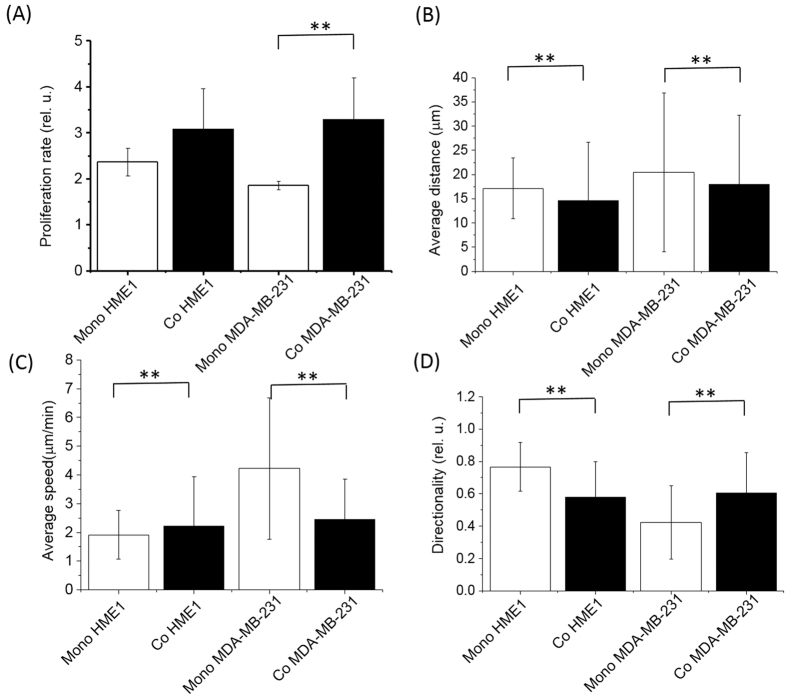
Proliferation rate and motility phenotypes of human breast normal epithelium (HME1) and metastatic breast adenocarcinoma (MDA-MB-231) cells under mono- and co-culture conditions. Compared with the BE cells, different behavior is observed. While proliferation rate (**A**) was slightly, but not significantly increased in the normal cells, the cancer cells proliferated significantly faster in co-culture with HME1 cells than in mono-culture (Table 2). All three motility parameters – distance, speed, and directionality – were altered significantly in both cell types under co-culture conditions (B, C, D, Supplementary Tables S4 and S5). While the average distance was slightly decreased in both cell types (**B**), differential response was observed in speed (**C**) and directionality (**D**). The speed of movement was decreased in HME1 cells, but increased in MDA-MB-231 cells. Directionality of HME1 cells decreased in co-culture as compared to a slight increase in MDA-MB-231 cells. Stars indicate statistical significance at p = 0.05.

To determine whether the findings of cell-cell communication characteristics in BE cells can be extended to a different tissue, we studied cellular communication that takes place between normal mammary epithelial HME1 and invasive ductal carcinoma cells MDA-MB-231. To this end, we co-cultured cells of the HME1 cell line with cells of the MDA-MB-231 cell line and performed the same type of analysis as with the co-cultured BE cells. Contrasting the finding in BE cells, MDA-MB-231 cells showed increased proliferation when co-cultured as compared to the mono-culture and the proliferation of HME1 cells did not show a significant difference between mono-culture and co-culture (Fig. 7A, Table 2). Cell motility analysis revealed a mixed picture of differential changes between the normal and breast adenocarcinoma cells (Fig. 7B–D, Supplementary Figure S4). The HME1 cells showed slight, but statistically significant alterations in their motility as a result of co-culture with MDA-MB-231 cells. Cell average speed was increased, but distance and directionality were decreased. The MDA-MB-231 cells exhibited a slight decrease in distance, a marked decrease in speed and an increase in directionality (Fig. 7B–D, Supplementary Figure S4, Supplementary Tables S4 and S5).

## Discussion

The findings of this study indicate that in the context of premalignant progression in BE, the role of normal epithelial cells is not limited to that of neutral bystanders. The presence of normal epithelium elicits a profound suppressive response in dysplastic BE cells. It appears that normal epithelium is able to not only “sense” the presence of abnormal (dysplastic, in this case) cells, but can also mount a drastic suppressive response via both direct cell-cell contact and soluble ligands. This finding is significant as it suggests that not only the immediate tumor microenvironment consisting of extracellular matrix, fibroblast cells and immune system cells, but also normal tissue surrounding a premalignant lesion can modulate its progression to cancer. It is possible that progression risk is determined at least in part by whether the suppressive action of the normal tissue can outbalance the growth of abnormal cells. This type of functional interaction between the two cell types may have significant implications in preventative and therapeutic strategies of cancer.

Our differential gene expression analyses have revealed TGFβ, EGF, and Wnt as key signaling pathways associated with the differential transcription profile observed in co-culture vs. mono-culture. While beyond the scope of this study, this finding warrants more targeted, in-depth inquiries into the molecular mechanisms associated with the enriched pathways underlying the suppressive effect of cellular communication at the transcriptional and protein level via e.g. analysis of post-transcriptional modifications of associated proteins. In what follows, we discuss several possible molecular mechanisms that may be at play based on the existing knowledge from previous published studies^17^,^18^,^19^,^20^,^21^.

TGFβ plays a dual role in carcinogenesis and cancer progression. In homeostasis and early stages of progression, it suppresses cell proliferation; in advanced stages, it functions as a promoter of cell proliferation, de-differentiation and extracellular matrix remodeling, while suppressing apoptosis^18^. TGFβ has been found to play a key role in the BE progression to EAC^17^. Our results show increased levels of inhibin βα (INHBA) in co-cultured dysplastic cells, which can suppress the TGFβ pathway through the TGFβ2 signaling arm^19^.

Suppression of the Wnt pathway resulted in significant inhibition of cell proliferation in esophageal squamous cell carcinoma cells^20^. It is thus likely that downregulation of expression of several Wnt members (Supplementary Figure S1) plays a role in suppressing growth and migration of dysplastic cells. Importantly, Wnt is closely associated with TGFβ resulting in crosstalk between the two pathways^21^. It is therefore possible that the observed down-regulation of the TGFβ signaling pathway is caused in part by the alterations in the Wnt signaling cascade.

The finding of significantly slower proliferation of the normal epithelial cells when co-cultured with dysplastic cells is interesting and suggests a strong mutual effect between the two cell types despite a seemingly smaller effect at the transcriptional level (234 vs. 809 statistically differentially expressed genes in normal and dysplastic cells, respectively). It is possible that regulation of cell proliferation takes place at the translational and/or post-translational level. Moreover, the finding of increased Euclidean distance and movement directionality of dysplastic cells in co-culture with normal cells suggests that the downregulation of the cell movement group at the transcriptional level (Fig. 2C) does not manifest as a functional phenotype and that motility may also be regulated at the protein level.

The finding of differential changes in the proliferation rate when comparing BE cells in co-culture and conditioned media is interesting and suggests that both mechanisms of cellular communication, secreted ligands and direct cell-cell contact, may be involved. Namely, if communication was mediated by secreted ligands only, the proliferation rate should be identical in conditioned media and co-culture. In this case, cells should experience the same culture conditions regardless of direct cell-cell contacts with the other cell type. On the other hand, if cellular communication took place exclusively via direct cell-cell contact, no difference should be observed between cells cultured in regular or conditioned media, where no direct cell-cell contact is present. Our data show that none of the two scenarios are true. First, compared with mono-culture, co-cultured CP-D cells proliferated at a markedly reduced (4.0-fold) rate (Fig. 4D, Table 1), whereas the proliferation rate increased 1.6 times in conditioned vs. regular media (Fig. 5A). Furthermore, EPC-2 cells proliferated 4.1 times slower in co-culture (Fig. 4D) compared to 1.27 times slower when in conditioned media (Fig. 6A, Table 1). This suggests that both communication mechanisms are at play in co-cultured normal and dysplastic BE cells. Furthermore, the differential proliferation response indicates that cell-cell contact between the cells of the two types is important as it can either enhance the suppressive effect of the soluble ligand communication in EPC-2 cells or reverse it from promoting to inhibiting in CP-D cells. Supporting the notion of importance of cell-cell communication via direct contact is the finding of inhibited cell proliferation in neoplastic rat liver cells when co-cultured with their normal counterparts that was dependent on the presence of direct cell-cell contact via gap junctions^22^. Loss of heterologous communication via gap junctions between non-tumorigenic and tumorigenic rat esophageal epithelial cells in the presence of extensive homologous communication^23^ provides further support for the importance of this type of cellular communication in cell homeostasis and normal function.

Addition of exogenous TGF-β1 or BMP7 showed similar effect on both cellular proliferation and motility. Both cytokines followed the same response pattern in the two BE cell types in conditioned and regular media (Figs 5 and 6). Because BMP7 has been demonstrated to antagonize TGF-β1 in the context of EMT, this finding suggests that in premalignant BE dysplasia the EMT transition and its associated signaling pathways may play a less important role as compared with EAC.

The finding of the differential effects of TGF-β1 and BMP7 between conditioned and regular media within the same cell type suggests that besides signaling via TGF-β1, other signaling mediators (e.g. EGF, Wnt, FGF) may be involved and responsible for the reversal of changes in the growth and motility phenotypes under the different incubation conditions (Figs 5 and 6).

The co-culture experiments conducted with malignant breast cancer cells of MDA-MB-231 cell line and their normal counterparts HME1 have shown that MDA-MB-231 cells proliferated faster in co-culture than in mono-culture, whereas HME1 cells did not show a difference in proliferation. This finding of a reverse effect of cellular communication in transformed breast cancer cells compared to premalignant BE cells suggests that cellular communication is tissue- and context-dependent. It is possible that due to the dysregulation or loss of gap junctional intercellular communication in transformed cancer cells^24^,^25^,^26^, soluble ligands may become the dominant mediator of cell-cell communication. The proliferation trend of cellular communication between MDA-MB-231 and HME1 cells appears similar to the effect observed with BE cells in conditioned media.

Cell motility analysis revealed (Fig. 7, Supplementary Tables S4 and S5) decreased distance and speed, but increased directionality of the MDA-B-231 cells, whereas the HME1 cells showed decreased distance and directionality, but increased speed. This finding suggests that, first, while cell proliferation of normal cells is not affected under co-culture conditions, cell motility undergoes slight but significant alterations. Second, the effect on motility is cell type-specific and differs from BE cells in co-culture. This finding warrants more in-depth studies focusing on particular signaling pathways identified in this study and/or additional candidates.

In summary, it is possible that in addition to transcription, other signaling mechanisms at the post-translational and/or protein level may play equally important or even pivotal functional roles. These types of interactions would be difficult to detect at the transcriptional level. We note that our data show substantial cellular heterogeneity in terms of proliferation and motility indicating a high degree of variability at the single cell level^27^,^28^ that underlies the observed changes in the ensemble averaged values. These results warrants more in-depth single cell studies targeted at the analysis of different sub-populations of cells with differing phenotypes. The findings of this study are important in that they reveal, for the first time, the effects of cellular communication on gene expression and cellular function between premalignant (dysplastic) epithelial cells and their normal counterparts. In early stages of disease, normal and abnormal epithelial cells often coexist *in vivo*, and the understanding of the functional role of interactions between them is of critical importance as it may help identifying new potent biomarkers for progression and for devising novel therapeutic strategies to prevent and/or reverse cancer progression.

## Materials and Methods

### Cell culture

CP-D cells were derived from a region of high-grade dysplasia of Barrett’s esophagus and hTERT immortalized. They were obtained from Dr. Maley’s lab at UCSF and authenticated by karyotyping in the same year. EPC-2 cells are hTERT immortalized human esophageal keratinocytes and were obtained from Dr. Rustgi’s lab at University of Pennsylvania and validated by karyotyping in the same year. The MDA-MB-231 cells were stained with 1 μM of CellTrace (far red, Invitrogen Cat. No. C34572). The HME1 cells were stained with 5 μM of CellTrace (CFSE, Invitrogen Cat. No. C34570). Both cell types were stained for 20 minutes at 37 °C. After staining, the cells were washed with Dulbecco’s Modified Eagle Medium. Then, the cells were trypsinized and put into a 24 well plate. Both cell lines were grown in Gibco Keratinocyte serum-free medium. Passage numbers of 15–25 after thawing from master vials were used to reduce the possibility of genetic drift.

### Cell culture in conditioned media, TGF-β1 or BMP7

The cells were trypsinized and then seeded at 7.5 × 10^4^ cells/well into each well of a 24-well plate. After 24 hours, the cells were stained with 1 μg/mL of Hoechst for 15 minutes at 37 °C. The medium was changed out with untreated, fresh keratinocyte growth medium and then the cells were imaged as part of the proliferation test. Directly after imaging, the cells were treated with their intended treatments which was one of the following: conditioned (other cell) medium, conditioned medium with BMP7, conditioned medium with TGF- β1, and regular medium. A second plate of EPC-2 and a second plate of CP-D cells were seeded at 7.5 × 10^4^ cells/well and the cell medium was removed from these cells and placed in the first 24-well plate (in respective wells) on a daily basis. After the cell medium was removed from the cells from the second plate, the cell medium was replenished in the second plate with fresh cell medium. For the following three days, the cells were imaged at a single time point with three images per well for the motility test.

A vial containing TGF-β1 (R&D Systems) was centrifuged prior to opening. Then the contents were reconstituted in 10 mM citric acid at pH 3.0, to a concentration of 50 μg/mL. The further dilution was made in PBS containing 2 mg/mL albumin so that the serum-free keratinocyte medium contained 5 ng/mL TGF-β1.

The BMP7 (Abcam, Catalog #ab50036) was reconstituted at 100 μg/mL in sterile 4 mM HCl containing at least 0.1% bovine serum albumin. The further dilution was made in PBS so that the serum-free keratinocyte medium contained 100 ng/mL BMP7.

### Next generation sequencing and differential gene expression analysis

Next generation sequencing was performed on an Illumina HiSeq 2000 sequencer with three replicates of each biological condition. BWA^29^ was used for genome alignment. Welch’s t-test, DESeq^9^ and EdgeR^10^ were used for differential gene expression analysis. Genes with logarithmic ratio of fold change ≥2 and FDR <0.05 (Benjamini-Hochberg correction) were identified as significant.

### Functional annotations and pathway enrichment

Functional and pathway analysis of statistically significant gene expression changes (candidate genes) were performed using Ingenuity Pathway Analysis (Qiagen, Redwood City, CA, USA). Fisher’s Exact Test was used to calculate p-values, which determined the probability of biological functions/pathways enriched in the candidate genes was due to the random effects. A Benjamini–Hochberg correction was performed for multiple testing corrections. Functions or pathways with p-values less than 0.05 were considered to be significantly relevant. The activation Z-score was calculated by using information about the direction of gene regulation. Comparison analysis of pathway enrichment from different statistical tests was also carried out using the Ingenuity Pathway Analysis.

### Time lapse fluorescent microscopy

Cells were stained with 1 μg/mL Hoechst 33342 (Life Technologies, Carlsbad, CA, USA) and incubated at 37 °C for 15 min. After 24 hours of co-culture, time lapse images were taken every 5 minutes for 1 hour using a Nikon Eclipse TE2000-E microscope (Nikon Inc., Melville, NY, USA). The microscope was equipped with a 20× phase contrast objective (Nikon Inc., Melville, NY, USA) and a cooled CCD camera (CoolSNAP HQ, Photometrics, Tucson, AZ, USA), controlled by NIS-Elements imaging software (Nikon Inc., Melville, NY, USA).

### Statistical analysis

Fisher’s exact test and Benjamini-Hochberg correction were performed to determine the significance of enriched functions. Z-scores of bigger than 2 and FDR of less than 0.05 were considered statistically significant.

Nonparametric Kolmogorov-Smirnov and t-test of statistical significance were performed using R language to determine significance of differences of cell motility and proliferation. P-values of less than 0.05 were considered statistically significant.

## Additional Information

**How to cite this article**: Zeng, J. *et al*. Transcriptional regulation by normal epithelium of premalignant to malignant progression in Barrett’s esophagus. *Sci. Rep.* **6**, 35227; doi: 10.1038/srep35227 (2016).

## Supplementary Material

Supplementary Information

Supplementary Table S1

Supplementary Table S2

Supplementary Table S3

Supplementary Table S4

Supplementary Table S5

Supplementary Movie S1

Supplementary Movie S2

Supplementary Movie S3

## Figures and Tables

**Table 1:**
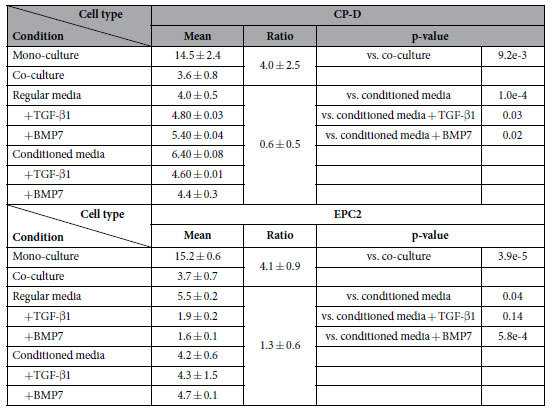
Differential changes in proliferation rates of normal esophageal epithelial (EPC-2) and dysplastic (CP-D) cells in co-culture and conditioned media.

The mean of rates were calculated as a ratio of the cell count at 96 vs. 24 hours of culture. “Ratio” represents a fold change in the proliferation ratio of cells under control and treatment conditions: mono-culture vs. co-culture, and regular vs. conditioned media, correspondingly. The p-values were calculated using the student’s t-test.

**Table 2:**
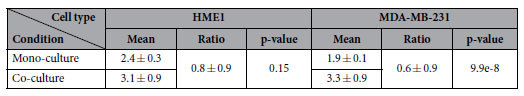
Proliferation rates of normal mammary epithelial (HME1) and metastatic breast adenocarcinoma (MDA-MB-231) cells under mono- and co-culture conditions.

The mean of rates were calculated as a ratio of the cell count at 96 vs. 24 hours of culture. “Ratio” represents a fold change in the cell proliferation rate of cells under mono- over co-culture conditions. The metastatic cells proliferated markedly faster in co-culture, the normal cells showed a slight, but statistically not significant increase in proliferation. The p-values were obtained using the student’s t-test.
