# Supplementary material for: Transcriptional regulation by normal epithelium of premalignant to malignant progression in Barrett’s esophagus
Source: Sci Rep. 2016 Oct 12;6:35227. doi: 10.1038/srep35227 (PMC5059688; doi:10.1038/srep35227)
Supplement: Supplementary Information [file srep35227-s1.pdf]

Supplementary Information

Title

Transcriptional regulation by normal epithelium of pre-malignant to malignant progression in Barrett's esophagus

Authors and affiliations

Jia Zeng[#1], Laimonas Kelbauskas[#*1], Aida Rezaie[1], Kristen Lee[1], Benjamin Ueberroth[1], Weimin Gao[1], Dmitry Derkach[1], Thai Tran[1], Dean Smith[1], Kimberly J. Bussey[1], and Deirdre R. Meldrum[1]

1. Center for Biosignatures Discovery Automation, The Biodesign Institute, Arizona State University, P.O. Box 876501, Tempe, AZ 85287-6501, United States

# - these authors contributed equally to the work

*Corresponding authors:

Laimonas Kelbauskas: Phone: +1 480-965-3128, Fax: +1 480-727-6588, E-mail: lkelbaus@asu.edu

Deirdre R. Meldrum: Phone: +1 480-727-9397, Fax: +1 480-965-2337, Email: deirdre.meldrum@asu.edu

Materials and Methods

Fluorescence assisted cell sorting of co-culture and mono-culture of normal and neoplastic cells

For CP-D and EPC-2 mono-cultures, 2×10^6 cells of each type were seeded into a 75 cm^2 flask. For CP-D and EPC-2 cells co-culture, 1×10^6 CP-D and 1×10^6 EPC-2 cells suspension were mixed and seeded into a 75 cm^2 flask. After 24 hours, cells were treated with 0.05% (v/v) trypsin–EDTA (Invitrogen, Carlsbad, CA, USA). The trypsinization was blocked by Dulbecco’s modified Eagle medium (DMEM) (Invitrogen, Carlsbad, CA, USA) supplemented with 5% fetal bovine serum (FBS) (Invitrogen, Carlsbad, CA, USA). After trypsinization, cells were centrifuged at 900 rpm for 3 min then re-suspended in 300 μL of PBS (Gibco, Carlsbad, CA, USA) and kept on ice. CP-D and EPC-2 cells were sorted out from co-culture on a BD FACS Aria (BD Biosciences, San Jose, CA, USA), using a 488 nm laser to excite *TurboGFP* in CP-D cells and *FP635* in EPC-2 cells. Manual compensation was performed to correct for spectral cross-over of fluorescent proteins. Mono-cultured CP-D and EPC-2 cells were also sorted using the same *TurboGFP* or *FP635* gates and regions.

RNA extraction

Total RNA was extracted from the sorted cells using RNeasy mini kit (Qiagen, Valencia, CA, USA) according to the manufacturer’s protocol. The silica-gel membrane and the spin-column technology removed the majority of the DNA. After proprietary buffer RW1 treatment, residual DNA in the RNA samples was digested on the column using RNase-free DNase set (Qiagen, Valencia, CA, USA) for 15 min at 20–30°C to remove DNA more completely. The RNase-free DNase set consists of 10 μL of DNase 1 stock solution in 70 μL of proprietary buffer

RDD. RNA was eluted by adding 30 µL of RNase-free water into the silica-gel membrane and stored at -80°C.

Whole transcriptome amplification

The quantity and purity of RNA obtained from FACS sorted cells was measured using spectrophotometry on a Nanodrop instrument (Agilent Technologies, Santa Clara, CA, USA). The concentration of RNA was adjusted to 10 ng/µL. 50 ng of RNA was amplified using Nugen Ovation RNA-Seq V2 kits (Nugen Technologies, San Carlos, CA, USA) per manufacturers' instruction on an Apollo 324 Library Preparation System (IntegenX, Pleasanton, CA, USA). Briefly, the RNA was reverse transcribed to the first-strand cDNA by using a combination of random hexamers and poly-T oligomer. The RNA template was fragmented by RNA-dependent DNA polymerase and double-stranded DNA was generated by the same polymerase. The dsDNA was amplified linearly using a single primer isothermal amplification (SPIA) process: RNase H degraded RNA in RNA in the DNA/RNA heteroduplex; the SPIA primer bound to the cDNA; the polymerase synthesized new cDNA strand replacing the RNA; random hexamers amplified the second-strand cDNA linearly. This technology covered non-coding RNA and non-polyadenylated RNA besides mRNA and reduced the conversion of ribosomal RNA to cDNA.

Amplified DNA was measured by a Nanodrop instrument for quality control purposes. cDNA was sheared to about 250 bp using a Covaris S2 instrument (Covaris, Woburn, MA, USA) and checked again using a Nanodrop instrument.

Cell lines

CP-D and EPC-2 cell lines were tagged with TurboGFP and TagFP635, respectively, to distinguish different cell types using fluorescence microscopy and fluorescence activated cell sorting. For lentiviral infection of cell lines, 1.3 x 104 CP-D cells and 1.6 x 104 EPC-2 cells were

seeded into individual wells of a Costar® 96-well-plate (Corning, Corning Life Sciences, Corning, NY, USA) containing 100 µL of Gibco Keratinocyte serum-free medium. After 24 hours of incubation, the cell culture reached about 80% confluency. 100 µL of Keratinocyte serum-free medium containing 8 mg/mL of hexadimethrine bromide was added to each well. Lentiviral MISSION® pLKO.1-puro-UbC-TurboGFP™ Positive Control Transduction Particles (Sigma-Aldrich, St Louis, MO, USA), containing a gene encoding TurboGFP under the control of the UbC promoter, were added to the well of CP-D cells at a multiplicity of infection of 2. Lentiviral MISSION® pLKO.1-puro-UbC-TagFP635™ Positive Control Transduction Particles (Sigma-Aldrich, St Louis, MO, USA), containing a gene encoding TagFP635 under the control of the UbC promoter, were added to the well of EPC-2 cells at a multiplicity of infection of 2 as well. The plate was gently stirred and centrifuged at 1000 rpm, 37° C for 30 minutes. After 18-20 hours, the medium containing lentiviral particles was replaced with 120 µL of Keratinocyte serum-free medium in each well. Four days after infection, the cells were imaged using a Nikon C1si (Nikon Inc., Melville, NY, USA) confocal microscope to inspect the expression of cytosolic TurboGFP in CP-D cells and TagFP635 in EPC-2 cells.

After the culture was expanded into 75 cm2 flasks (Corning, Corning, NY), a puromycin kill curve experiment was performed to determine the minimum concentration of puromycin to cause 0% viability ration in puromycin treated cells. 1.0 µg/mL and 0.5 µg/mL of puromycin were found to effectively kill the CP-D and EPC-2 cells, respectively, in which TurboGFP or TagFP635 was not successfully expressed after 96 hours. CP-D and EPC-2 cells were grown in keratinocyte serum-free medium containing puromycin (1.0 µg/mL for CP-D cells and 0.5 µg/mL for EPC-2 cells) for five passages, and then grown in normal keratinocyte serum-free

medium. The expression of cytosolic TurboGFP or TagFP635 was retained in CP-D and EPC-2 cells, respectively, when checked under a Nikon C1si confocal microscope.

Library preparation and Illumina sequencing

Illumina sequencing libraries were prepared on an Apollo 324 Library Preparation System using the PrepX™ ILM DNA Library Preparation kit (IntegenX, Pleasanton, CA, USA) with four different barcoded adapters for multiplexing. The adapter-ligated libraries were amplified by 10 cycles of PCR using a KAPA HiFi Library Amplification Kit (Kapa Biosystems, Woburn, MA, USA). PCR amplified libraries were qualified using high sensitivity DNA assay on an Agilent Bioanalyzer 2100 (Agilent Technologies, Santa Clara, CA, USA) and quantified using a KAPA Library Quantification Kit - Illumina/Universal (Kapa Biosystems, Woburn, MA, USA).

Clusters were generated using the cBot platform (Illumina, San Diego, CA, USA). Four samples were multiplexed per lane with two lane replicates. Single-end sequencing with 50 base reads was performed on an Illumina HiSeq 2000 (Illumina, San Diego, CA, USA) following the manufacturer's guidelines. Four samples were multiplexed per lane with two lane replicates on an Illumina HiSeq 2000 sequencer.

Next-generation sequencing alignment

The sequenced reads were parsed based on the index to allow analysis of the data on the individual sample basis. Raw reads were analyzed using the GeneSifter® Analysis Edition pipeline (PerkinElmer, Inc., Seattle, WA, USA), a cloud-based software architecture. After quality assessment, raw reads were aligned to the *Homo sapiens* genome reference build 37.2 (GRCh37.p2) using Burrows-Wheeler Aligner [66] with Genome Analysis Toolkit [67] for variant calling. From approximately 100 million single-end 50-bp sequencing reads, a median of 72

million reads per sample were mapped to the reference. The majority of mapped reads are annotated gene features (exon-intron regions) and rRNA or snRNA, followed by intergenic regions.

Differential gene expression

Three methods were used for identifying differential gene/transcript expressions:

1) In the GeneSifter pairwise analysis pipeline, the raw read count was normalized by total mapped million reads (RPM) and reported as log2 values. Welch's t-test (does not require equal variance between two groups) was run on the log transformed RPM between two conditions to test whether transcript levels were changed due to intracellular interactions. A Benjamini–Hochberg correction was performed for multiple testing adjustments. Genes were considered as differentially expressed when logarithmic ratio of fold change ≥ 2 and false discovery rate (FDR) < 0.05.
2) DESeq[1] of R/Bioconductor[2], an analysis based on the negative binomial distribution, was also used for differential expression analysis. The count values from different samples were normalized to the library size factors so that they were on a common scale. Genes with logarithmic ratio of fold change ≥ 2 and FDR < 0.05 (Benjamini-Hochberg correction) were declared significant.
3) EdgeR package [3] of R/Bioconductor , another testing based on negative binomial model, was used for differential analysis as well. The raw count data was normalized using trimmed mean of M-values (TMM) between each pair of samples as the scale factors. After the inter-library dispersions were estimated, an exact test was performed to identify differentially expressed genes. Genes with logarithmic ratio of fold change ≥ 2 and FDR < 0.05 (Benjamini-Hochberg correction) were identified as significant.

Venn diagrams showing the overlaps of gene candidates from four different statistical tests were drawn using Venn Diagram Plotter (PNNL; http://omics.pnl.gov).

RNA extraction, reverse transcription and qPCR

Quantitative real-time PCR was used to validate changes in gene expression. Primers for each of the target sequences were selected from PrimerBank [4] or designed using the Primer-BLAST tool (*www.ncbi.nlm.nih.gov/tools/primer-blast/*). Multiple primer pairs were designed and evaluated at the bulk cell RNA level. Optimized primer oligos were obtained from Integrated DNA Technologies (Integrated DNA Technologies, Coralville, IA, USA).

RNA extracted from FACS sorted co-culture and mono-culture cells was used for reverse transcription and qPCR. A total volume of 20 µL of the cDNA synthesis mixture contained the following reagents: 4 µL of 5 × VILO Reaction Mix (Invitrogen, Carlsbad, CA, USA), 2 µL of 10 × SuperScript Enzyme Mix (Invitrogen, Carlsbad, CA, USA), including SuperScript III RT, RNaseOUT Recombinant Ribonuclease Inhibitor, and a proprietary "helper" protein (Invitrogen, Carlsbad, CA, USA), and 14 µL of total RNA. The contents in each tube were gently mixed and spun down, and the cDNA synthesis was performed in following thermal steps: (a) 25 °C for 10 min, (b) 42 °C for 60 min, and (c) 85 °C for 5 min to inactivate the reverse-transcriptase. The cDNA obtained from these reactions was stored at -20 °C until further use.

Prior to qPCR, cDNA was diluted 500 times by adding DEPC-treated water (Ambion, Austin, TX, USA). The qPCR runs were conducted using the following reagent mixtures: 5 µL of EXPRESS SYBR GreenER qPCR SuperMix Universal (Invitrogen, Carlsbad, CA, USA), 1 µL of each primer (4 µM), 0.1 µL of ROX Reference Dye (25 µM) (Invitrogen, Carlsbad, CA, USA), 2 µL of diluted cDNA, and 0.9 µL of DEPC-treated water (Ambion, Austin, TX, USA). For negative controls, 2 µL of DEPC-treated water was used instead of cDNA. The thermal

cycling profile was set up as follows: one cycle at 95 °C for 10 min; 40 cycles consisting of 95 °C for 15 s, 60 °C for 1 min, and 80 °C for 10 s with signal detection; melt-curve analysis at 60 °C for 1 min and the temperature increased in 0.3 °C increments to 95 °C, then at 95 °C for 15 s. The experiments were run on a StepOne Real Time PCR System (Applied Biosystems, Carlsbad, CA, USA). Data analysis was carried out using the StepOne software (Applied Biosystems, Carlsbad, CA, USA). *28S* were used to normalize samples for comparison.

Cell culture for time lapse fluorescent microscopy

To create the CP-D and EPC-2 cells co-culture, 3.75×10^4 CP-D and 3.75×10^4 EPC-2 cells suspension were mixed and seeded into individual wells of a Costar® 24-well-plate (Corning Life Sciences, Corning, NY, USA). For CP-D and EPC-2 mono-culture, 7.5×10^4 cells of each type were seeded into individual wells in the 24-well-plate. Cells were cultured for 24 hours before time lapse fluorescent microscopy.

Image analysis

Images were analyzed using custom-written MATLAB (MathWorks Inc., Natick, MA, USA) code and a Fiji TrackMate plugin 70.

Supplementary Figures, Tables and Videos

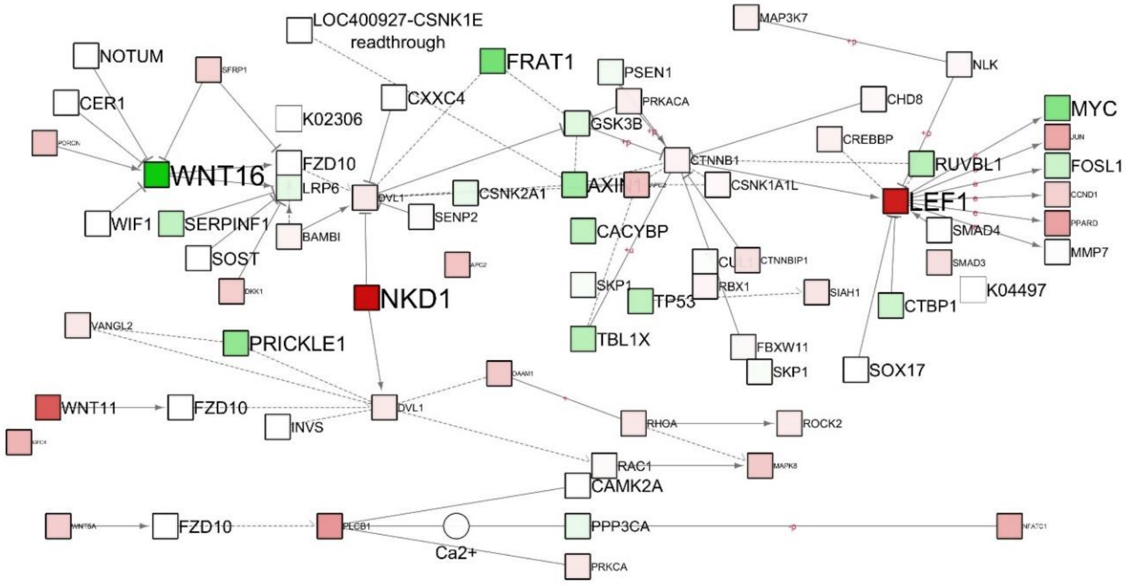

Figure S1. Wnt-signaling pathway is enriched in the differentially expressed genes between cCP-D cells in co-culture with EPC-2 cells and mono-culture. Red: upregulated in co-cultured CP-D cells. Green: down-regulated in co-cultured CP-D cells.

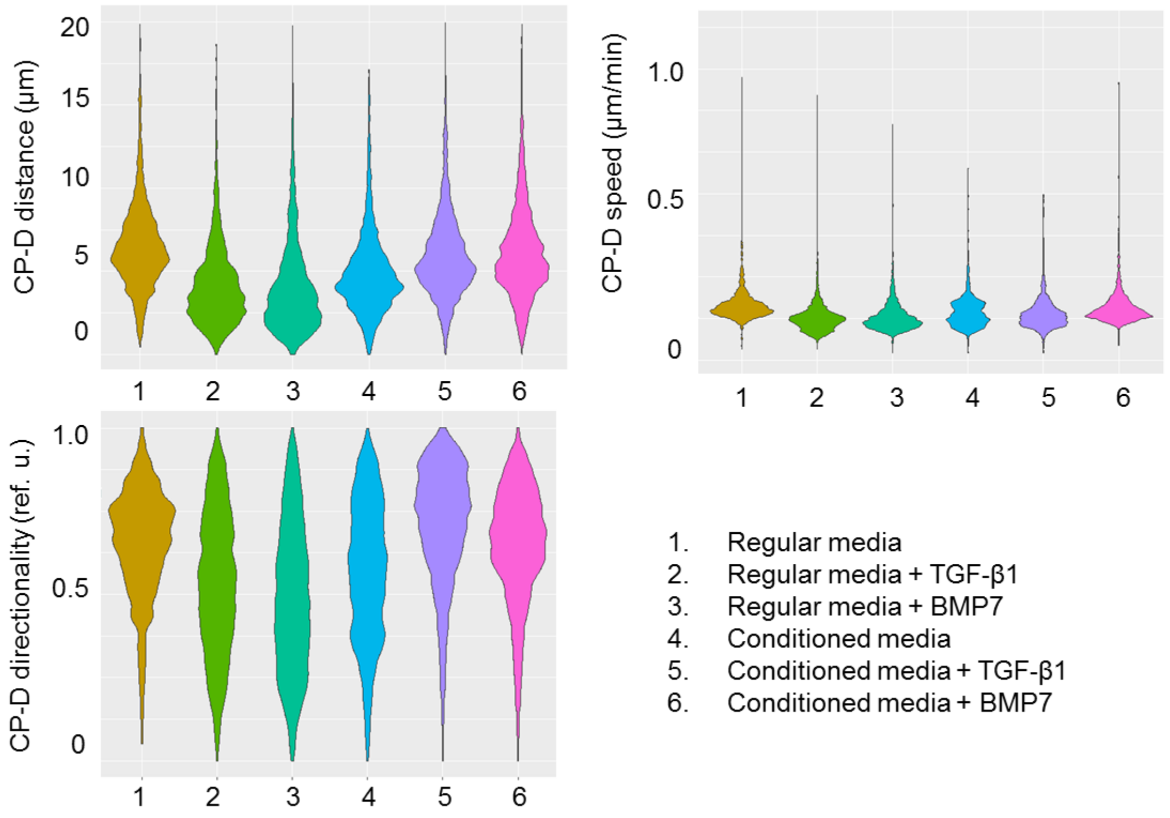

Figure S2. Violin plots of CP-D cell motility characteristics

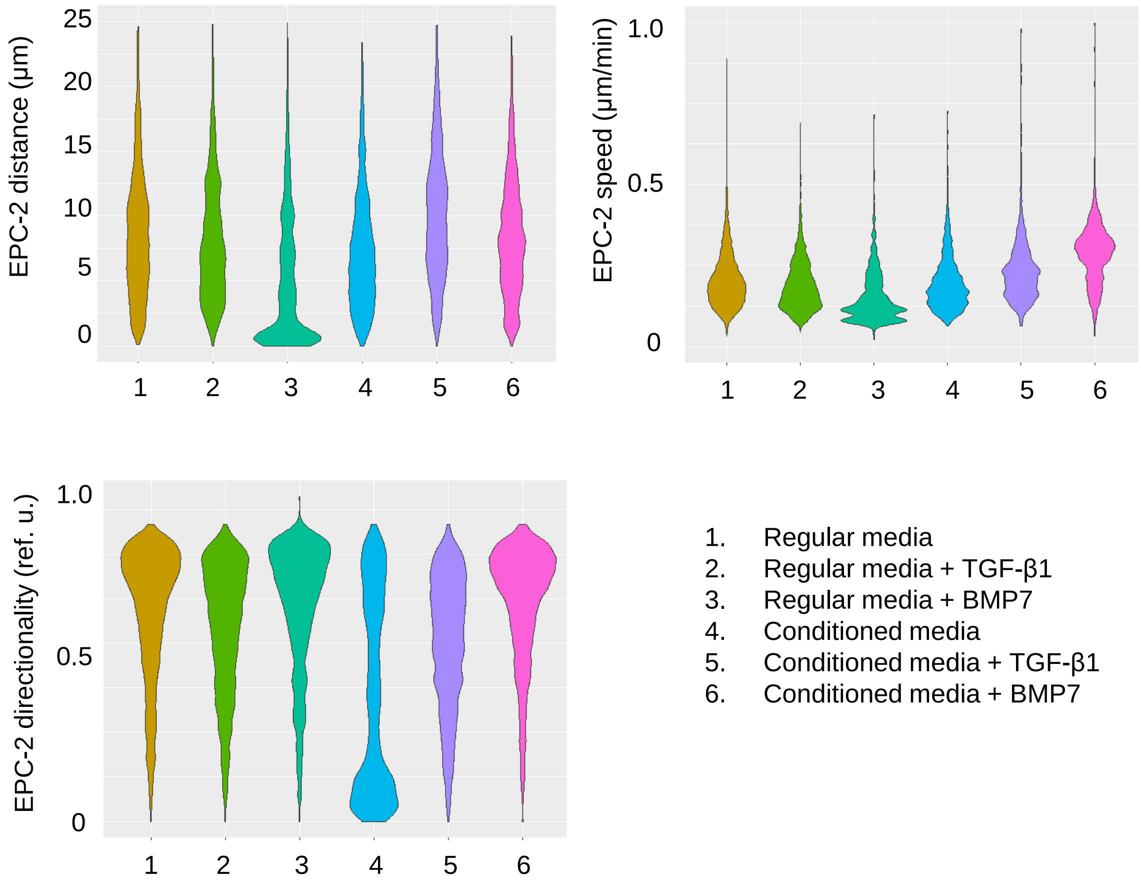

Figure S3. Violin plots of EPC-2 cell motility characteristics

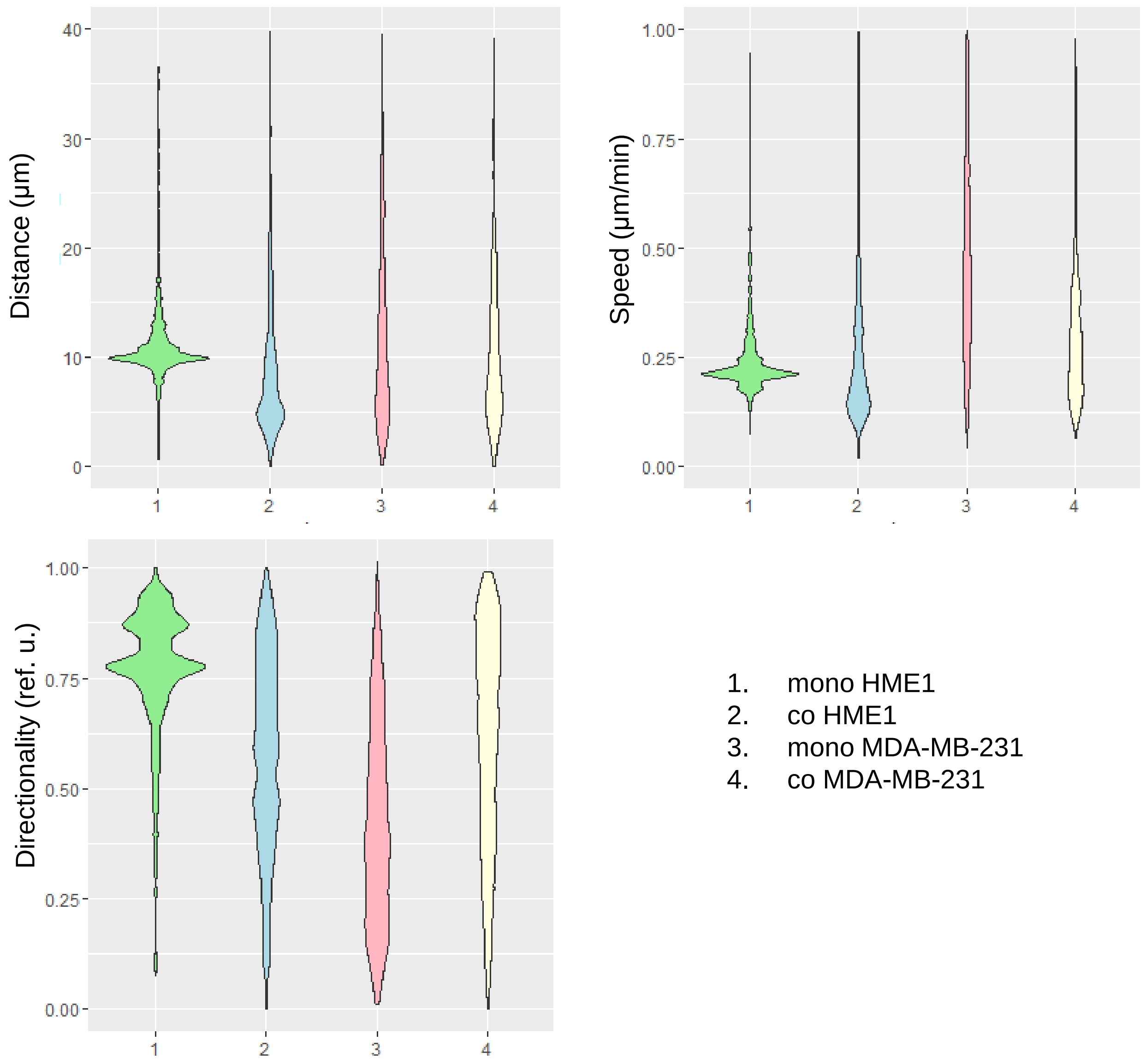

Figure S4. Violin plots of HME1 and MDA-MB-231 cell motility

Table S1

qPCR validation of RNA-Seq expression level measurements (Excel spreadsheet).

Table S2

Differentially expressed genes identified by all three tests - DESeq, EdgeR and welch's t-test (Excel spreadsheet).

Table S3

Mean values of CP-D and EPC-2 motility parameters (Excel spreadsheet). The error is presented as standard deviation.

Table S4

Mean values of HME1 and MDA-MB-231 motility parameters (Excel spreadsheet). The error is presented as standard deviation.

Table S5

P values for CP-D, EPC-2, HME1 and MDA-MB-231 motility using Kolmogorov–Smirnov test (Excel spreadsheet).

Movie S1

Representative video of mono-culture CP-D cell motility. Scale bar: 50 μm.

Movie S2

Representative video of mono-culture EPC-2 cell motility. Scale bar: 50 μm.

Movie S3

Representative video of co-culture of CP-D and EPC-2 cells motility. Scale bar: 50 μm.

References

1 Anders, S. & Huber, W. Differential expression analysis for sequence count data. *Genome Biol.* **11**, R106, (2010).

2 Gentleman, R. C. *et al.* Bioconductor: open software development for computational biology and bioinformatics. *Genome Biol.* **5**, R80, (2004).

3 Robinson, M. D., McCarthy, D. J. & Smyth, G. K. edgeR: a Bioconductor package for differential expression analysis of digital gene expression data. *Bioinformatics* **26**, 139-140, (2010).

4 Wang, B., Wood, I. S. & Trayhurn, P. PCR arrays identify metallothionein-3 as a highly hypoxia-inducible gene in human adipocytes. *Biochemical and biophysical research communications* **368**, 88-93, (2008).
